# Kaposi’s Sarcoma Lesion Progression in BKV-Tat Transgenic Mice Is Increased by Inflammatory Cytokines and Blocked by Treatment with Anti-Tat Antibodies

**DOI:** 10.3390/ijms23042081

**Published:** 2022-02-14

**Authors:** Egidio Brocca-Cofano, Cecilia Sgadari, Orietta Picconi, Clelia Palladino, Antonella Caputo, Barbara Ensoli

**Affiliations:** 1 Department of Chemical, Pharmaceutical and Agricultural Sciences, University of Ferrara, Via Fossato di Mortara 64B, 44121 Ferrara, Italy; ebrocca@bluespherebio.com; 2 BlueSphereBio, University of Pittsburgh, 350 Technology Drive, Suite 520, Pittsburgh, PA 15219, USA; 3 National HIV/AIDS Research Center, Istituto Superiore di Sanità, Viale Regina Elena 299, 00161 Rome, Italy; cecilia.sgadari@iss.it (C.S.); orietta.picconi@iss.it (O.P.); clelia.palladino@iss.it (C.P.)

**Keywords:** HIV-1 tat, BKV/Tat transgenic mice, KS-like lesions, inflammatory cytokines, anti-Tat antibodies, KS regression, KS progression

## Abstract

Kaposi’s sarcoma (KS) is an angioproliferative tumor showing an increased frequency and aggressiveness in HIV-infected subjects (AIDS-KS), due to the combined effects of inflammatory cytokines (IC), angiogenic factors, and the HIV-1 Tat protein. While the introduction of effective combined antiretroviral regimens greatly improved AIDS-KS incidence and course, it continues to be an incurable disease and the development of new rational targeted therapies is warranted. We used the BKV/Tat transgenic mouse model to evaluate the effects of IC and anti-Tat antibodies (Abs) treatment on KS-like lesions arising in BKV/Tat mice. We demonstrated here that IC-treatment increases the severity and delays the regression of KS-like lesions. Further, anti-Tat Abs reduced KS-like lesion severity developing in IC-treated mice when anti-Tat Abs were administered at an early-stage of lesion development as compared to more advanced lesions. Early anti-Tat Abs treatment also accelerated KS-like lesion regression and reduced the rate of severe-grade lesions. This effect was more evident in the first weeks after Ab treatment, suggesting that a longer treatment with anti-Tat Abs might be even more effective, particularly if administered just after lesion development. Although preliminary, these results are encouraging, and the approach deserves further studies for the development of anti-Tat Ab-based therapies for AIDS-KS. Clinical studies specifically addressing the effect of anti-Tat antibodies in treating AIDS-KS are not yet available. Nevertheless, the effectiveness of anti-Tat antibodies in controlling HIV/AIDS progression, likely due to the neutralization of extracellular Tat activities, is suggested by several cross-sectional and longitudinal clinical studies, indicating that anti-Tat Ab treatment or Tat-based vaccines may be effective to treat AIDS-KS patients or prevent the tumor in individuals at risk.

## 1. Introduction

Kaposi’s sarcoma (KS) is an angioproliferative tumor first described in elderly men of the Mediterranean Eastern-European areas (classical KS) and also observed in patients treated with chronic immunosuppressive therapy, especially in organ transplant recipients (iatrogenic KS), and in children and young/adults of subequatorial Africa (African KS) [1]. In recent decades, this tumor has gained notoriety due to its increased occurrence in HIV-infected patients (AIDS-KS) [1,2]. Albeit the introduction of combined antiretroviral therapies (cART) has markedly changed the incidence and clinical course of AIDS-KS, this tumor remains a significant burden of morbidity and mortality, especially in sub-Saharan Africa, where it represents the first and second most common cancer in men and women, respectively, due to limited drug availability and cART adherence [2,3]. Moreover, relapses or refractory cases with an aggressive and often fatal course, which were frequently seen in the pre-cART era, are still observed today, particularly as an immune reconstitution inflammatory syndrome (IRIS) occurring only a few weeks or months after cART initiation [3].

All epidemiological forms of KS generally arise in the skin of the extremities as multiple patches, plaques, or nodular lesions. The tumor shows a particularly aggressive behavior when it is associated with HIV infection, with frequent involvement of mucosae and visceral organs [1]. At histology, early lesions (patch or plaque) appear as a granulation-type reaction infiltrated by immune cells and characterized by intense angiogenesis and proliferating spindle-shaped cells of endothelial and macrophage cell origin, that are considered to be the tumor cells of KS (KS cells) [1,3,4,5]. In late (nodular) lesions, KS cells eventually become the predominant cell type and lesions acquire a fibrosarcoma-like aspect, although neoangiogenesis remains always evident [1].

Previous studies indicated that, at least in early stages, KS is a cytokine-mediated disease and that inflammatory cytokines (IC) and angiogenic factors cooperate in its induction [1]. Specifically, IC such as interferon-γ (IFN-γ), tumor necrosis factor-α (TNF-α), interleukin (IL)-1, and IL-6 are increased in lesions and blood of KS patients and in individuals at risk of KS even before lesion development [6,7,8,9,10,11]. In these patients, IC are produced by activated blood mononuclear cells and by tissue-infiltrating T cells and monocytes/macrophages [12,13], perhaps in response to (or amplified by) infection of human herpesvirus-8 (HHV-8) [1,3,12,13,14,15] that is considered the etiologic agent of KS, although its presence is not sufficient for disease development [1,15].

The early-stage lesions have a polyclonal nature and can regress [1,15,16,17]. However, over time they can become monoclonal, especially in the nodular stage, and can evolve into a true sarcoma, probably in association with the increased expression of HHV-8 and anti-apoptotic oncogenes [1,15,18,19,20,21].

In this context, the Tat protein of HIV-1 acts as a progression factor in AIDS-KS and increases the frequency and aggressiveness of AIDS-KS as compared to other non-HIV associated epidemiological KS forms [1,15]. In particular, extracellular Tat, released during acute infection of T cells by HIV-1 [22,23], promotes the growth, migration, invasion, and adhesion of KS cells, activated endothelial cells and monocytes [22,24,25,26]. Tat can also transactivate, in both infected and uninfected cells, the expression of cellular genes coding for cytokines, chemokines, or their receptors, thus enhancing the inflammatory milieu that promotes HHV-8 replication and KS development by a paracrine mechanism [1,22]. In addition to this, Tat enhances HHV-8 infectivity in endothelial cells, promotes the recruitment of HHV-8-infected circulating KS cells from blood, and potentiates the angiogenic and oncogenic activity of HHV-8 proteins such as vGPCR, vIL-6, K1, and kaposin A through the modulation of the PI3K/AKT pathway [15,27,28,29,30,31,32].

Interestingly, transgenic mice carrying the *tat* gene develop dermal lesions resembling the early phase of KS [33,34]. In particular, mice carrying a transgene containing the BK virus (BKV) early-region and the HIV-1 *tat* gene, driven by its own promoter (the HIV-1 LTR) (BKV/Tat transgenic mice), develop dermal lesions like the early phases of KS, as well as tumors of different histotype also observed in HIV-positive persons [34,35].

The dermal lesions appear around the third month of life in 40% of male mice and are characterized by squamous cell dermal hyperplasia and hyperkeratosis localized in the back [34,35]. These lesions are generally mild, present skin thickening followed by scabbing and localized ulceration. Only sporadically they assume a more severe course with localized lesions merging in large, ulcerated area often associated with hemorrhages. The lesions last generally 1 to 2 months and eventually regress, as sometime observed in patients, and even the animals with more severe lesions can return to normality [34,35].

At histology, the bed of the ulcers appears to be formed by granulation tissue with a prominent capillary network, infiltration by inflammatory cells (macrophages, lymphocytes, and leukocytes), and proliferation of spindle-shaped cells and endothelial cells, simulating the histopathological features of early KS lesions [34,35].

Of note, spindle cells isolated from ulcerated KS-like skin lesions of BKV/Tat-transgenic mice co-express antigens specific for endothelial, smooth muscle, and antigen-presenting cells, and express a complex mixture of angiogenic factors, including fibroblast growth factor-2 and vascular endothelial growth factor [36], as observed in human KS lesions and primary KS cells isolated from human lesions [1,24,37].

Thus, many pathological findings observed in this animal model, including KS-like skin lesions, resemble the lesions appearing in AIDS patients, suggesting a relevant role for extracellular Tat in the pathogenesis of such lesions during the course of AIDS.

Altogether, these data suggest that BKV/Tat transgenic mice represent a good model to investigate the pathogenic role of extracellular Tat in AIDS-KS development as well as anti-AIDS-KS interventions. Here, we used this model to evaluate the effects of IC and anti-Tat antibodies (Abs) treatment on KS-like lesions’ development and progression.

## 2. Results

To elucidate the role of IC and extracellular Tat in KS lesion development and progression, we evaluated the effects of IC (IL-1*β*, TNF-*α*, and IFN-γ) and anti-Tat Abs on KS-like lesions developing in BKV/Tat transgenic mice.

**Figure 1:**
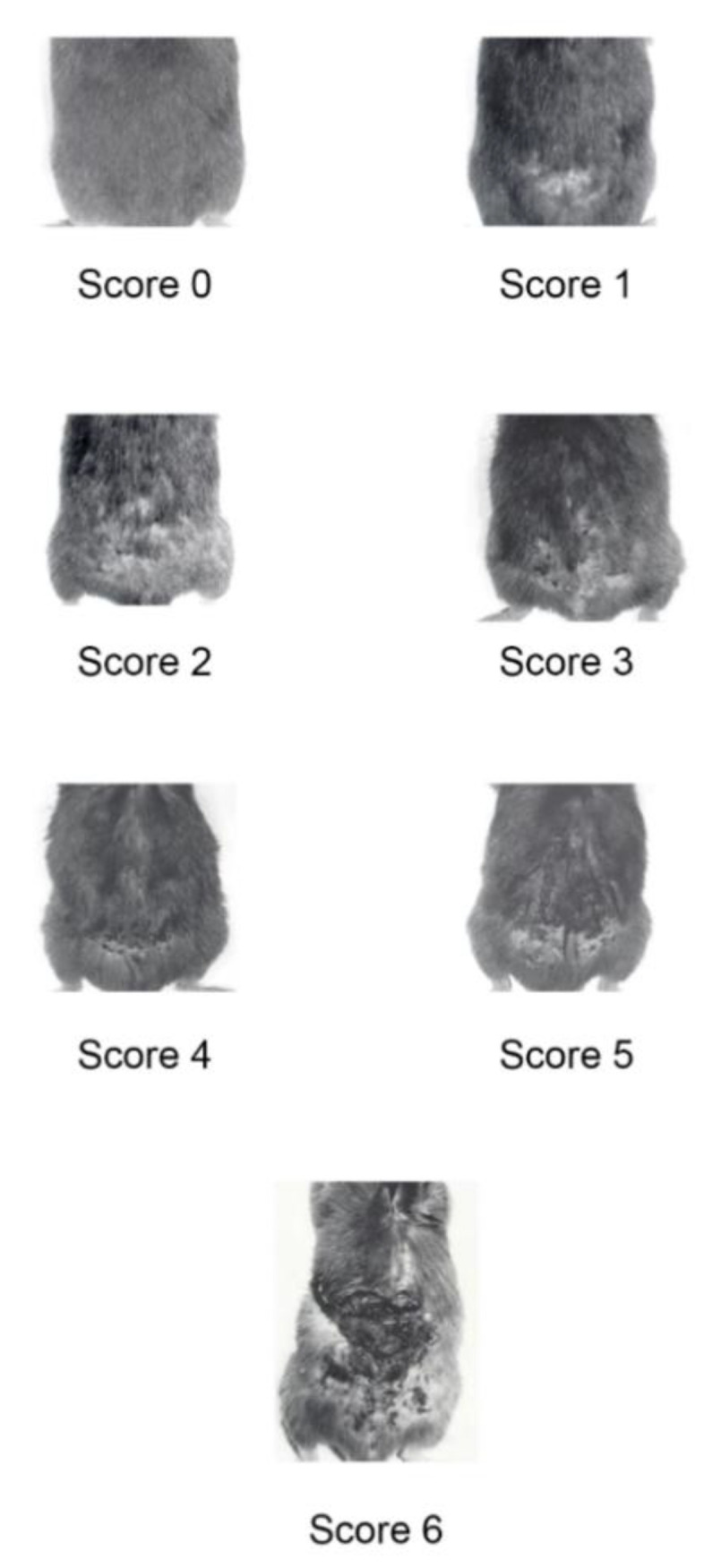
KS-like lesions developing at the dorsal area of BKV/Tat transgenic male mice, scored according to lesion severity, as described in Methods.

To this aim, BKV/Tat mice bearing initial KS-like lesions or no lesion were injected locally with a mixture of IL-1, TNF-*α*, and IFN-*γ* three times (day 0, 4, 8) and were monitored up to 10 weeks as compared with untreated mice. Lesion dynamic was evaluated by grading lesion severity according to the scoring system depicted in Figure 1.

**Figure 2:**
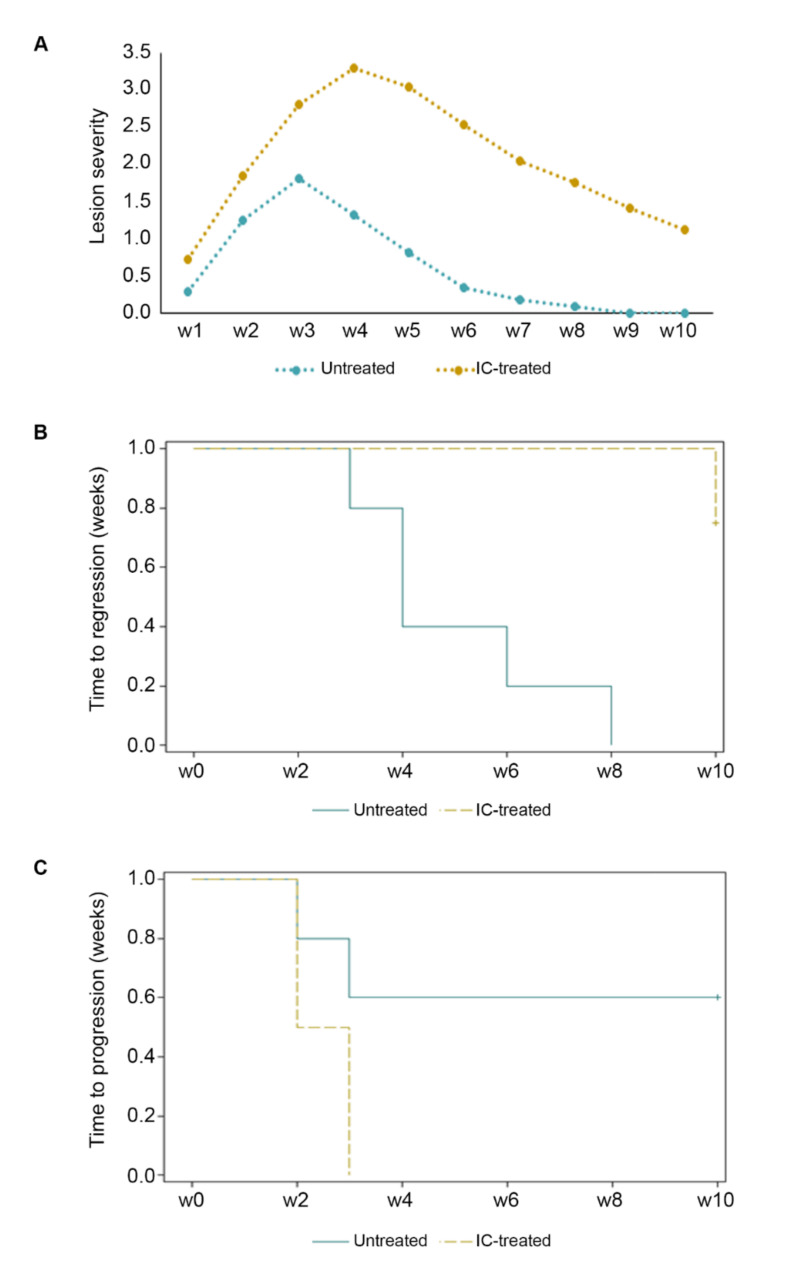
Increase in lesion severity and duration after IC-treatment as compared to untreated lesions in BKV/Tat transgenic mice. BKV/Tat transgenic mice bearing initial lesions or no lesions were treated with IC at 0, 4, and 8 days as described in Methods (4 mice) (yellow line), or were only observed (5 mice) (teal line). Mice were monitored twice/week and lesion severity scored up to week 10 as described in Methods and as depicted in Figure 1. (**A**) Mean lesion severity over time in IC-treated versus untreated mice (ANOVA for repeated measures, between subjects effects *p* = 0.0054, and interaction effects = 0.0393). (**B**) Time to regression (weeks) to a severity score consistently ≤ 0.5, assessed by Kaplan–Meier method (Log-Rank test, *p* 0.046). (**C**) Time to progression (weeks) to a severity score ≥ 3, evaluated by Kaplan–Meier method (Log-Rank test, *p* = 0.0779).

In IC-treated mice, lesion severity and course over time were significantly worse as compared to untreated controls (Figure 2A). Moreover, lesions of IC-treated mice regressed significantly slower as compared to untreated controls (Figure 2B), where they disappeared completely by week 8, while IC-treated mice still presented a severity score > 1 by the end of the observation time (week 10) (Figure 2A). In addition, lesions of IC-treated mice tended to progress to a score ≥ 3 faster as compared to untreated mice (Figure 2C). Of note, when the skin lesions were already present at the beginning of the IC treatment, they tended to progress faster and to regress slower as compared to mice without lesions at the beginning of the observation (Figure S1).

In order to assess the role of extracellular Tat on lesion course, mice were divided into two groups: IC-treated mice bearing lesions with a score of ≤0.5 were defined as early-stage, whereas mice with lesions of ≥1 were defined as advanced-stage or late-stage. Both groups were injected with IC at day 0, 4, and 8 and anti-Tat Abs or control Abs at day 4, 8, and 12. Lesion course was monitored up to week 12.

**Figure 3:**
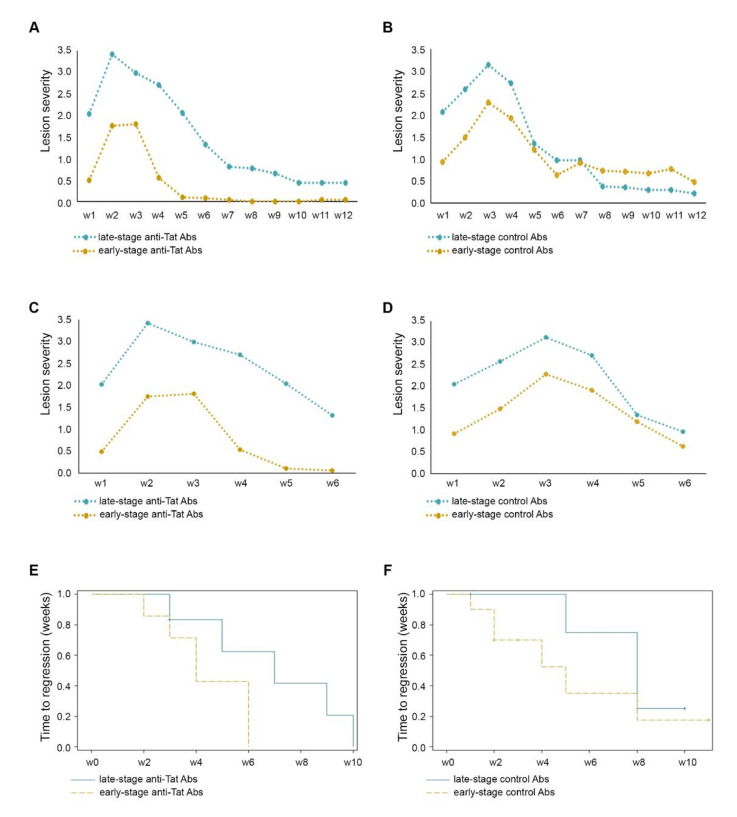
Effects of anti-Tat Abs in early or advanced IC-treated KS-like lesions of BKV/Tat transgenic mice. IC-treated (day 0, 4, 8) mice with early-stage (score ≤ 0.5) or late-stage (score ≥ 1) KS-like lesions were inoculated with anti-Tat Abs or control Abs (day 4, 8, 12) (early-stage mice: yellow line; late-stage mice: teal line). Mice were monitored twice/week and lesion severity was scored up to week 12. (**A**) Mean lesion severity over time in anti-Tat Abs injected mice with early-stage (7 mice) (yellow line) or late-stage (teal line) (8 mice) KS-like lesions. ANOVA for repeated measures, between subjects’ effects *p* = 0.0594. However, when the analysis was limited at the first 6 weeks (**C**), the between subjects’ effects was *p* = 0.0170. (**B**) Mean lesion severity over time in control Abs-injected mice with early-stage (10 mice) (yellow line) or late-stage (teal line) (5 mice) KS-like lesions. No differences were detected by ANOVA for repeated measures when the analysis was performed up to 12 weeks (**B**) or 6 weeks (**D**). Time to regression (weeks) to a severity score consistently ≤ 0.5 in early stage versus late-stage lesions treated with anti-Tat Abs (**E**) versus control Abs (**F**) assessed by Kaplan–Meier method (Log-Rank test, *p* = 0.0734).

Notably, in the group of mice in which the anti-Tat Ab treatment was started at an early stage, the lesion score tended to be lower as compared to the group of mice in which the anti-Tat Abs were given at a more advanced stage (Figure 3A), while no difference was observed in the lesion course of control mice (Figure 3B). In particular, the greater effect of anti-Tat Abs in early-stage versus late-stage lesion bearing-mice was more evident in the first weeks after anti-Tat Ab treatment, when it reached a statistical significance (ANOVA for repeated measures *p* = 0.0170) (Figure 3C), while no difference was detected for the same timeframe in control Abs-injected animals (Figure 3D). Although anti-Tat treated mice with advanced-lesions showed a similar course as compared to control-Ab mice, the scoring of lesions in mice treated with anti-Tat Abs at an early-stage tended to be less severe as compared to control-Ab mice, not only in the first weeks from the anti-Tat Ab treatment but also at later time points (Figure 3A–D).

In addition, 100% (7/7) of the mice treated with anti-Tat Abs at an early-stage regressed to a lesion score stably ≤ 0.5 by week 6, while at the same time point only 50% (5/10) of early-stage control mice reached the same degree of regression. This threshold was never reached by 40% (4/10) of control-Ab mice when the whole observation period was considered (lesion score range: 0.8–6). Finally, the lesions of mice treated at an early stage tended to regress more rapidly as compared to the lesions of mice treated in a more advanced stage (Log-Rank test, *p* = 0.0734) (Figure 3E), while no difference was observed in control mice (Figure 3F). Of note, one out of seven animals treated at an early stage showed progression to a score ≥3 as compared to those treated later (14% versus 75%, respectively), while the early-stage and late-stage mice treated with control Abs showed a similar progression rate (40% versus 60%, respectively) (Table S1).

Altogether, these results indicate that anti-Tat Ab treatment may be effective against KS-like lesions of BVK/Tat transgenic mice when administered at early-stages of lesion development.

## 3. Discussion

We have shown here that treatment with the same IC that are increased in lesions and blood of KS patients or in subjects at risk of disease development enhances the severity and delays the regression of KS-like lesions arising in BKV/Tat transgenic mice.

This observation agrees with preclinical and clinical data indicating that IC play an important role in the development of all KS forms [1,15]. In particular, it was demonstrated that IC induce in endothelial cells the functional features and the biomarkers of KS spindle cells, such as the spindle-cell morphology, the downregulation of factor VIII-related Ag expression, and the up-regulation of adhesion-molecules expression [13,38,39]. In addition, similarly to KS cells, endothelial cells activated by IC become angiogenic in nude mice [13,38,39,40]. This is because IC induce production and release of angiogenic factors such as basic fibroblast growth factor (bFGF) and vascular endothelial growth factor (VEGF), which are highly expressed in all forms of KS and synergize in promoting angiogenesis, matrix-metalloproteases expression and activation, vascular permeability, and edema [13,37,38,39,40,41,42,43] as observed in human KS lesions [1,15].

Of note, IC or bFGF are required to observe extracellular-Tat activity either in, in vitro or in vivo models of AIDS-KS, including endothelial and KS cell proliferation, migration, invasion, matrix-metalloproteases expression, and angiogenesis [13,24,44,45,46,47,48]. This is because they induce endothelial cells to express the receptors for Tat, identified as the α5β1 and αvβ3 integrins, which are constitutively expressed by KS cells [25,44,45,46]. Specifically, the RGD sequence present at the carboxyl terminus of Tat binds these receptors and mediates the migration, invasion, and adhesion of KS cells and IC-activated endothelial cells [25,44,45]. Thus, Tat mimics the action of extracellular-matrix molecules such as fibronectin and vitronectin that bind to the same receptors [25] and thus provides endothelial cells with the adhesion signal they require to grow. These mechanisms are likely to be operative in vivo since bFGF and Tat are both present in AIDS-KS lesions and Tat co-stains with β1 and β3 integrins on resident vessels and spindle cells [46].

Interestingly, IC-induced integrins also function as receptors for HIV and HHV8 in several cell types, including dendritic cells and activated endothelial cells [49,50,51,52], thus favoring viral infection and spread. In this context, HIV-1 Tat and several HHV-8 encoded proteins, such as vIL-6 and vGPCR, contribute to establishing an inflammatory and angiogenic environment favorable to tumor progression through the collaborative stimulation of the PI3-K/Akt/GSK-3 pathway activity [28,30]. In addition, studies conducted in vitro and in patients showed that Tat positively modulates the expression of IC with immunosuppressive activity, including IL-10 [53,54,55,56,57,58], while HHV8-encoded proteins such as vIRF and LANA promote the immune escape by regulating the host responses (reviewed in [59]), indicating that the dysregulation of the immune system is a mechanism by which these viruses contribute to AIDS-KS progression, which, however, appears to be a later event.

Altogether, these data indicate that Tat and IC synergize in promoting KS development and progression and that IC-treated BKV/Tat transgenic mice represent a suitable model to test interventions against AIDS-KS, although it does not allow the evaluation of HHV8-promoted oncogenesis.

By using our transgenic model, we found that three doses of anti-Tat Abs reduced KS-like lesion severity developing in IC-treated BKV/Tat transgenic mice when anti-Tat Abs were administered at an early-stage of lesion development. Early anti-Tat Abs injection also accelerated KS-like lesion regression and reduced the rate of severe-grade lesions. This effect was more evident in the first weeks after Ab treatment, suggesting that a longer treatment with anti-Tat Abs might be more effective, particularly if administered just after lesion development.

Previous data obtained in, in vitro and in vivo models of KS and angiogenesis indicated that anti-Tat Abs can neutralize the activity of extracellular Tat [24,46,60]. The results described here, confirm and extend those studies by using an in vivo model of Tat-induced, IC-promoted KS-like lesions that more closely resemble the microenvironment leading to AIDS-KS. Although preliminary and with some limitation due to the small sample of transgenic mice tested here, these results are encouraging and the approach deserves further studies for the development of anti-Tat Ab-based therapies for AIDS-KS, a tumor that despite recent improvements continues to be an incurable disease also on the cART era [2]. Interestingly, antiretroviral drugs included in cART such as HIV protease inhibitors, which have been shown to block angiogenesis and tumor cell invasion and to induce tumor cell apoptosis and growth arrest [61,62,63], are now under evaluation alone or associated with chemotherapy in KS and other tumors, in HIV-infected or seronegative patients [64,65]. Of note, the advances made in understanding the mechanisms underlying KS pathogenesis led to the recent design and evaluation of rational targeted therapies, such as agents inhibiting angiogenesis and/or multiple cellular pathways [66].

Although a clinical translation specifically addressing the effect of anti-Tat Abs in treating AIDS-KS is not available now, observational and interventional studies by our and other groups (extensively reviewed in [67]), indicating that the presence of anti-Tat antibodies and anti-Tat immunity are effective in controlling HIV/AIDS progression, suggest a protective role of anti-Tat antibodies due to the neutralization of extracellular Tat activities. In this context, anti-Tat Ab treatment or Tat-based vaccines may be effective to treat AIDS-KS patients or prevent the tumor in individuals at risk.

## 4. Materials and Methods

### 4.1. BKV/Tat Transgenic Mice Monitoring and Treatment

The generation of BDF1 transgenic mice carrying BKV/tat sequences, crossing with outbred CD1 mice and subsequent breeding were previously described [34,35,36]. At 2-month age a tail fragment was taken by male mice to confirm the presence of the *tat* transgene by polymerase chain reaction (PCR). Briefly, the tail sample was incubated at 50 °C for 14–16 h in lysis buffer (50 mM tris(hydroxymethyl)aminomethane hydrochloride (Tris-HCl)) pH8, 100 mM Ethylene Diaminetetraacetic Acid (EDTA), 100 mM sodium chloride, 1% Sodium Dodecyl Sulfate, 500 ug/mL proteinase K), DNA extracted by phenol-chloroform and analyzed (200–300 ng) by PCR reaction (50 uL) using Tat-specific primers (Tat-Forward: 5′GAAGCATCCAGGAAGTCAGCC 3′; Tat-reverse: 5′ACCTTCTTCTTCTATTCCTTCGGG3′) (1 mM each), 50 uM dNTPs, TaqI DNA polymerase (1 U) and 1X Taq I buffer (94 °C for 5 min; 35 cycles: 94 °C for 30 s, 55 °C annealing for 30 s, 72 °C TaqI polymerization: 72 °C extension for 10 min; 4 °C for cooling down for 10 min). Samples were then controlled by gel electrophoresis.

Transgenic mice were monitored twice/week for physical and behavioral changes, and examined for KS-like lesion development, which typically appears on the lower dorsal area around 3/4-months age in 40% of male mice [34,35].

KS-like lesion severity (Figure 1) was graded according to the following score. Score 0: no lesion; score 0.5: slight hair loss and skin thickening; score 1: mild alopecia with superficial scabbing; score 2: mild alopecia with deeper scabbing; score 3: marked alopecia and sparse skin ulceration; score 4: skin ulceration with some exposition of deeper derma and muscle layer; score 5: large, confluent ulcerated skin areas with exposed muscle layer; score 6: deep, large ulceration with detachment of the derma from the underlying tissues.

To assess the effect of cytokines on development/regression of the lesions, mice with early-stage lesions (score ≤ 0.5) were treated with a cocktail of IC (IL-1*β*, IFN-*γ*, TNF-*α*) at days 0, 4, and 8. Inflammatory cytokines (murine IL-1*β* (0.2 µg/mouse), IFN-*γ* (0.5 µg/mouse), TNF-*α* (0.5 µg/mouse), Roche Diagnostics, S.p. A., Monza, MB, Italy) were combined in a volume of 225 µL, mixed with an equal volume of matrigel (Becton Dickinson, BD Biosciences Discovery Labware, Bedford MA, USA) and administered subcutaneously in the lower back of the animals. Untreated mice with initial lesions (score ≤ 0.5) were used as control. All mice were observed for lesion score twice a week up to week 10.

To determine the effect of IC and Tat-Abs on the development/regression of the lesions, mice were divided into two experimental groups: mice with early-stage lesions (score ≤ 0.5) and mice with late-stage lesions (score ≥1). Both groups were treated with IC at days 0, 4, and 8, and then with anti-Tat Abs or with a control rabbit serum (control groups) at days 4, 8, and 12. Rabbit anti-Tat polyclonal antibodies (Intracel Corporation, Issaquah, WA, USA) or control rabbit polyclonal IgG (Sigma-Aldrich, St. Louis, MO, USA) were inoculated subcutaneously in the lesion (100 µL). All mice were observed for lesion score twice a week up to week 12.

### 4.2. Statistical Analysis

The weekly lesion-score was calculated as the mean of 2-weekly lesion-score evaluation. ANOVA for repeated measures was performed in order to compare the lesion score between groups.

Kaplan–Meier method was used to assess time to regression, intended as a score consistently ≤ 0.5, and time to progression, intended as the first time of a score ≥ 3.0 and groups were compared by the Log-Rank test. Statistical analyses were carried out using SAS^®^ (Version 9.4, SAS Institute Inc., Cary, NC, USA).

## Data Availability

The data presented in this study are available on request.

## Supplementary Materials

The following supporting information can be downloaded at: https://www.mdpi.com/article/10.3390/ijms23042081/s1.
