# Supplementary material for: Kaposi’s Sarcoma Lesion Progression in BKV-Tat Transgenic Mice Is Increased by Inflammatory Cytokines and Blocked by Treatment with Anti-Tat Antibodies
Source: Int J Mol Sci. 2022 Feb 14;23(4):2081. doi: 10.3390/ijms23042081 (PMC8874961; doi:10.3390/ijms23042081)
Supplement: Supplementary file 1 [file ijms-23-02081-s001.zip › ijms-1544352-supplementary.pdf]

Supplementary materials

Figure S1. Time course of single-lesion severity in IC-treated mice with measurable KS-like lesions or no lesions.

BKV/Tat transgenic mice bearing initial lesions or no lesions were treated with IC at 0, 4, 8 days as described in Methods (4 mice). The Figure shows the single lesion severity over time of the IC-treated mice already presented in Figure 2.

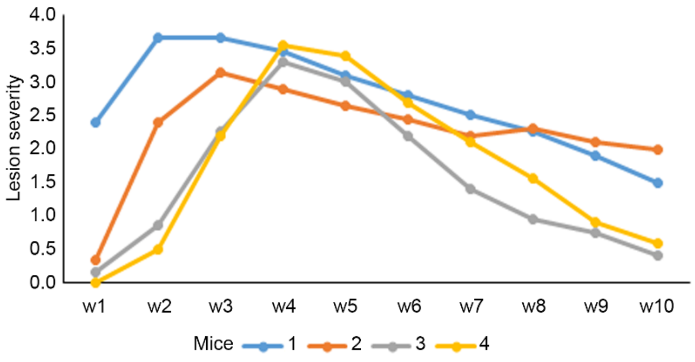

Table S1. Early-stage versus late-stage anti-Tat Ab treated lesions progressing to a score ≥3 over the monitoring course.

	n/N	%
**Early-stage anti-Tat Ab treatment**	1/7	14.29
**Late-stage anti-Tat Ab treatment**	6/8	75.00
**Early-stage control-Ab treatment**	4/10	40.00
**Late-stage control-Ab treatment**	3/5	60.00

IC-treated (day 0, 4, 8) mice with early-stage (score ≤ 0.5) or late-stage (score ≥ 1) KS-like lesions were inoculated with anti-Tat Abs or control Abs (day 4, 8, 12). Mice were monitored twice/week and lesion severity scored up to week 12. The Table shows the number and percentage of mice progressed to a score ≥3 over the monitoring course.
